# GnRH agonist early follicular challenge test as a predictor of ovarian response in antagonist cycles for fertility preservation

**DOI:** 10.1038/s41598-024-65059-4

**Published:** 2024-06-21

**Authors:** Gil M. Yerushalmi, Sarit Avraham, Alon Kedem, Michal Youngster, Jonathan Barkat, Ohad Baruchin, Itai Gat, Odelia Yaakov, Yariv Gidoni, Ariel Hourvitz

**Affiliations:** 1 IVF Unit, Department of Obstetrics and Gynecology, Shamir Medical Centre, Tzrifin, affiliated with the Faculty of Medical and Health Science, Tel Aviv University, Tel Aviv, Israel; 2 Alan and Ada Selwyn Chair for Clinical Infertility Research and Molecular Medicine, Faculty of Medical and Health Science, Tel Aviv University, Tel Aviv, Israel

**Keywords:** Fertility preservation, Ovarian reserve test, GAST, Assisted reproduction, Predictive markers, Endocrine reproductive disorders

## Abstract

The aim of our study was to evaluate if the response to follicular GnRH agonist (GnRHa) trigger be used to predict intracycle ovarian response in GnRH antagonist cycles among women undergoing fertility preservation IVF. We conducted a prospective study of 146 GnRH antagonist oocyte pickup (OPU) cycles to evaluate GnRHa stimulation test (GAST). On day 2 of the cycle, basal E2 were measured, followed by injection of 0.2 mg GnRHa as part of the initial ovarian stimulation. 12 h later blood sampling was repeated (GAST E3). E2 response was used as test parameter. The major outcome was the number of mature cryopreserved oocytes. We found a linear correlation between both GAST E3 level and GAST E3/E2 ratio and number of M2 oocytes. ROC curve analysis of GAST E3, GAST E3/E2 ratio, AFC and day 3 FSH for > 15 M2 and < 5 M2 oocytes was calculated. For GAST E3 levels obtaining < 5 M2 oocytes, an AUC value of 0.79 was found. For GAST E3 levels obtaining > 15 M2 oocytes, AUC value of 0.8. Patients with GAST E3 ≤ 384 pmol/l has 58.6% risk to obtain < 5 oocytes. Patients younger than 35 with GAST E3 > 708 pmol/l have 66% chance for freezing > 15 oocytes. The response to single GnRHa administration during GnRH antagonist cycle can be used as biomarker of ovarian reserve. This simple, widely available marker, which reflect the estradiol response of small follicles, might predict the response of the specific cycle, and can potentially be used to adjust the treatment dose.

Trial registration number: 0304-20-ASF.

## Introduction

Over the past decade the rate of fertility preservation cycles has dramatically increased following the development of oocyte vitrification techniques. In the USA there was over 20-fold increase in the past decade to 23,898 fertility preservation cycles in 2020^1,2^.

The success of oocyte cryopreservation (OC) is not guaranteed and the chance of live birth after thawing is affected by many factor especially the age of the patient at the time of the OC procedure and the number of cryopreserved oocytes^3^.

It is desirable to have 15–20 oocytes available in patients aged < 35 years and 25–30 oocytes in women aged > 38 to achieve reasonable success rates^3^. Several studies have shown that young poor responder achieve poorer outcome in terms of oocyte survival and cumulative live birth rate when compared to normal responders^4^.

Ovarian reserve testing to predict response to gonadotropins stimulation is superior to the use of chronological age alone^5–7^. Currently, the antral follicle count (AFC), Anti Mullerian hormone (AMH) levels and basal FSH levels are most commonly used to estimate ovarian reserve^8^.

The ovarian response to GnRH agonist (GnRHa) administration (GAST test) is considered a valid marker for ovarian reserve^9,10^. A two-fold rise or more in estradiol (E2) concentration in response to GnRHa has been shown to be predictive of IVF success^9,10^. GAST was mostly evaluated in poor response, high risk for cancelation population. The reported dose for this test was 0.1 mg, however, from the vast experience with GnRH agonist triggering, it is now agreed that the optimal dose for GnRH agonist flare efect is 0.2 mg^11^.

The positive impact of the initial surge in hormones triggered by GnRH agonists (flare-up effect) during ovarian stimulation for follicular recruitment is well-established, specifically in short GnRH agonist protocols and ultra-short antagonist protocols^12^.

It was claimed that GAST was not superior to other tests^13^ but the test was never validated for short antagonist protocol. Moreover, its validation for the specific cycle was not tested.

In the current study we evaluated the use of GAST in short antagonist protocol as part of the social fertility preservation cycle. This study was part of another study that sought to predict suboptimal response to GnRH agonist triggering in fertility preservation cycles^14^. GAST was assessed as a predictor for the number of mature oocytes that are suitable for cryopreservation.

## Results

A total of 146 patient undergoing social fertility preservation cycle completed the study protocol. Table 1 represents parameters related to patient demographic as well as GnRHa stimulation test (GAST) hormonal results, treatment characteristics (gonadotropin dose and days of stimulation) and the ovarian response.

**Table 1:** Demographics and clinical treatment characteristics in study group.

	Mean ± std. error (range) (N = 146)
Age (years)	35.02 ± 2.53 (21–40)
G (Gravida)	0.13 ± 0.46 (0–3)
P (Para)	0.04 ± 0.23 (0–2)
Weight (kg)	62.63 ± 11.50 (45–110)
Height (cm)	163.00 ± 6.54 (145–180)
Body Mass Index	23.58 ± 4.07 (17–37)
Baseline LH (IU/l)	5.56 ± 2.674 (0.8–22)
Baseline E2 (pmol/l)	153.30 ± 53.57 (27–268)
Baseline FSH (IU/l)	7.29 ± 2.86 (2.6–18.5)
Antral Follicle Count (AFC)	12.87 ± 0.59 (3–30)
Duration of stimulation (days)	9.27 ± 1.90 (6–15)
Day 2 estradiol concentration (pmol/l)	153.30 ± 58.81 (27–268)
Day 3 estradiol concentration (pmol/l)	698.76 ± 369.75 (27–1529)
Day 3/Day 2 estradiol ratio	4.68 ± 2.41 (1–12.6)
Day 2 LH concentration (IU/l)	7.22 ± 2.77 (0.4–19.6)
Day 3 LH concentration (IU/l)	52.44 ± 19.62 (7.5–120)
Day 3/Day 2 LH ratio	9.17 ± 19.16 (1.42–236.75)
Total amount of gonadotropins (IU)	2757.25 ± 984.66 (1050–6600)
Gonadotropin dose per day (IU)	296.29 ± 74.47 (137.5–450)
E2 at trigger (pmol/l)	11,214.27 ± 7769.79 (1430–48,963)
Progesterone at trigger (pmol/l)	2.86 ± 1.78 (0.3–9.67)
LH at trigger (IU/l)	2.54 ± 9.62 (0.2–4.93)
FSH at trigger (IU/l)	21.01 ± 7.38 (7.9–47.5)
M1 oocytes no	1.03 ± 1.21 (0–5)
M2 oocytes no	11.10 ± 8.16 (0–48)
GV oocytes no	1.54 ± 2.17 (0–11)
Total no. oocytes	13.68 ± 9.48 (0–58)

In order to analyze the correlation between GAST E3 absolute levels and GAST E3/E2 relative levels to ovarian response, oocytes number we first used a linear regression analysis. In addition, used logistic regression to identify factors associated with poor response (less than five oocytes) and high response (more than 15 oocytes). Then, a ROC analysis of these parameters, including AFC and basal day 3 FSH as a predicting test for poor and high response. Finally, we used decision tree CHAID (Chi-square automatic interaction detector) analysis to automatically classify GAST E3 levels in poor and high responders.

Using linear regression, we found that the ratio between estradiol levels 12 h after GnRHa administration (GAST E3) and baseline estradiol levels (E2) positively correlated with the number of mature oocytes (M2 oocytes) suitable for cryopreservation (r = 0.61). Similarly, GAST E3 levels alone had also good positive correlation to the number of M2 oocytes (r = 0.58). An increase of 1% in GAST E3/E2 ratio correlate with 0.92% increase in M2 oocytes, adjusted to age. Accordingly, an increase of 1% in GAST E3 absolute value correlate with 0.82% increase in M2 oocytes, adjusted to age.

We performed multi variable regression analysis that included GAST E3, GAST E3/E2 ratio and the patient’s age. We used logistic regression to identify the groups of M2 ≤ 5 oocytes compared to M2 > 5 oocytes. It was found that the patient’s age was not significantly associated to predict the number of M2 oocytes obtained. The gonadotropin daily dose negatively correlated to the number of M2 oocytes. This is probably due to the fact that suspected low ovarian reserve patients were treated with higher dose of gonadotropins without a significant effect on M2 oocytes obtained.

We evaluated the prognostic value of GAST to predict the retrieval of more than fifteen M2 oocytes or less than five M2 oocytes using ROC curves analysis. We compared it to established ovarian reserve markers AFC and basal day 3 FSH levels.

**Figure 1:**
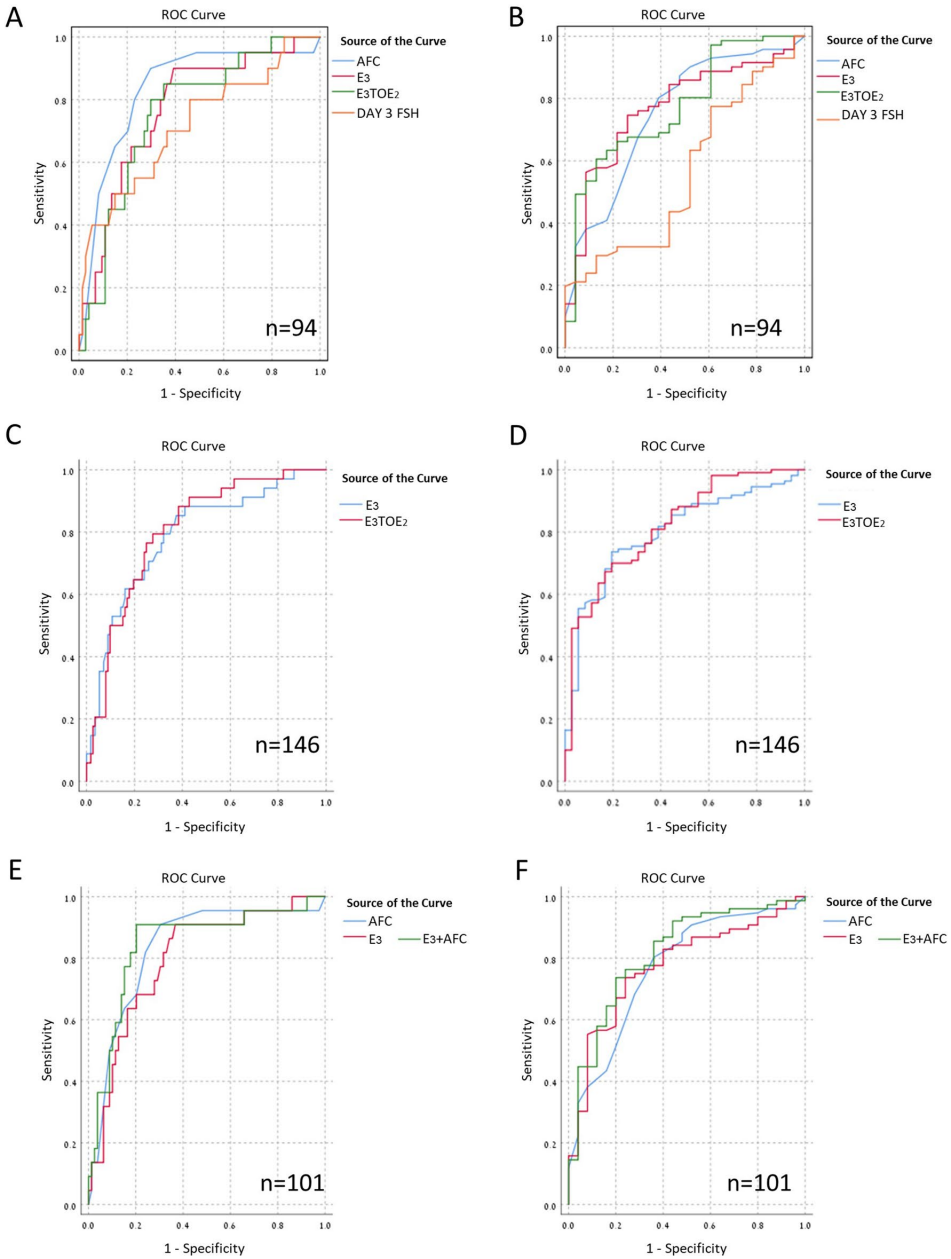
ROC curve for the relationship levels of different variables and the retrieval of ≤ 5 M2 oocytes or > 15 M2 oocytes. ROC curves comparing the efficacy of AFC, GAST E3, GAST E3/E2 and day 3 FSH for the prediction of ≤ 5 M2 (**A**) and > 15 M2 (**B**) oocytes. ROC curves for GAST E3 vs. GAST E3/E2 for the prediction of ≤ 5 M2 (**C**) and > 15 M2 (**D**) oocytes. ROC curves for AFC, GAST E3 and combined GAST E3+AFC models for the prediction of ≤ 5 M2 (**E**) and > 15 M2 (**F**) oocytes.

As shown in Fig. 1A and B, AFC, day 3, FSH, GAST E2/E2 and GAST E3, all markers achieved high AUC value indicating good prognostic value for the test to predict high response as well as poor response.

We compared each pair of the tests (all combinations of the four analyzed tests) and found that GAST E3, GAST E3/E2 and AFC had similar prognostic value to predict more than fifteen M2 oocyte or less than five M2 oocytes while Day 3 FSH had a significantly lower AUC value (Table 2).

**Table 2:** ROC analysis for prediction of M2 oocyte yield according to different factors.

	M2 ≤ 5				M2 > 15			
	AUC	Cutoff	Sensitivity	Specificity	AUC	Cutoff	Sensitivity	Specificity
AFC	0.83	9.5	68%	79%	0.77	14.5	64%	80%
Day 3 FSH (IU/l)	0.66	8.34	82%	50%	0.64	5.47	46%	79%
GAST E3 (pmol/l)	0.79	598	85%	64%	0.81	711	74%	80%
GAST E3/E2	0.81	3.68	72%	79%	0.82	4.55	81%	70%
GAST E3+AFC	0.85	n/a	90%	80%	0.82	n/a	74%	80%

Optimal Sensitivity and specificity were determined according to Youden’s index.

When examining GAST E3 levels we calculated AUC value of 0.80 (p < 0.0001) for over fifteen M2 oocytes with cutoff value of 711 pmol/l (Sensitivity 74% and specificity 81%) (Fig. 1D). We calculated AUC value of 0.79 (p < 0.0001) for obtaining less than five M2 oocytes with cutoff value of 598 pmol/l (Sensitivity 85% and specificity 62%) (Fig. 1C).

Combining AFC+GAST E3 to predict treatment outcome (for M2 ± 5 Z = 2.478–0.183*AFC−0.003*E3 and for M2 > 15 Z = − 4.52 + 0.133*AFC + 0.002*E3) resulted in non-significant higher AUC in ROC analysis compared to AFC or GAST E3 alone (0.85 for M2 ≤ 5 and 0.81 for M2 > 15) (Fig. 1E and F).

All ROC analysis results for all parameters are presented in detail in Table 2.

Based on the ROC analysis cutoff values, we analyzed the group of patients according to GAST E3 above and below 598 pmol/l (presented at Table 3) and revealed that those with GAST E3 below 598 pmol/l were not older but presented with significantly higher day 3 FSH (although mean FSH was within normal values), lower AFC, treated with higher doses of gonadotropins per day, achieved lower E2 levels on ovulation triggering and attained significantly less mature oocytes suitable for cryopreservation (Table 3). The group of patients exhibiting GAST E3 above 711 pmol/l were not younger but presented with lower day 3 FSH, higher AFC and needed a lower dose of gonadotropin dose per day (Table 4). These results strengthen the prognostic value of GAST to predict the ovarian response.

**Table 3:** Demographics and clinical treatment compared between patients GAST E3 cutoff value of 598 pmol/l.

	GAST E3 < 598 (N = 71)	GAST E3 ≥ 598 (N = 75)	p-Value
AGE (years)	35.10	34.94	0.5
BMI	24.15	22.99	0.21
Baseline E2 (pmol/l)	138.75	167.85	0.001
Baseline FSH (IU/l)	8.40	6.2	< 0.001
AFC	10.78	14.75	< 0.001
Duration of stimulation (days)	9.70	8.84	0.001
Gonadotropin dose per day (IU)	324.26	269.05	< 0.001
E2 at trigger (pmol/l)	8358.40	13,974.97	< 0.001
M2 oocytes	7.25	14.73	< 0.001

**Table 4:** Demographics and clinical treatment compared between patients GAST E3 cutoff value of 711 pmol/l.

	GAST E3<711 (n = 88)	GAST E3≥711 (n = 58)	p-Value
AGE (years)	35.15	34.82	0.23
BMI	23.88	23.07	0.32
Baseline E2 (pmol/l)	144.38	167.73	0.006
Baseline FSH (IU/l)	8.01	6.14	< 0.001
AFC	11.21	15.51	< 0.001
Duration of stimulation (days)	9.47	8.93	0.09
Gonadotropin dose per day (IU)	317.54	262.73	< 0.001
E2 at trigger (pmol/l)	8574.24	12,925.21	< 0.001
M2 oocytes	7.77	16.16	< 0.001

Examining the subgroup of patients in which less than 5 mature oocytes were obtained we identified 17 patients (11.6% of the cohort, 47% of patients with less than 5 M2 oocytes) with apparently normal day 3 FSH levels (less than 10.3 IU/l) but with low GAST E3 (less than 598 pmol/l).

**Figure 2:**
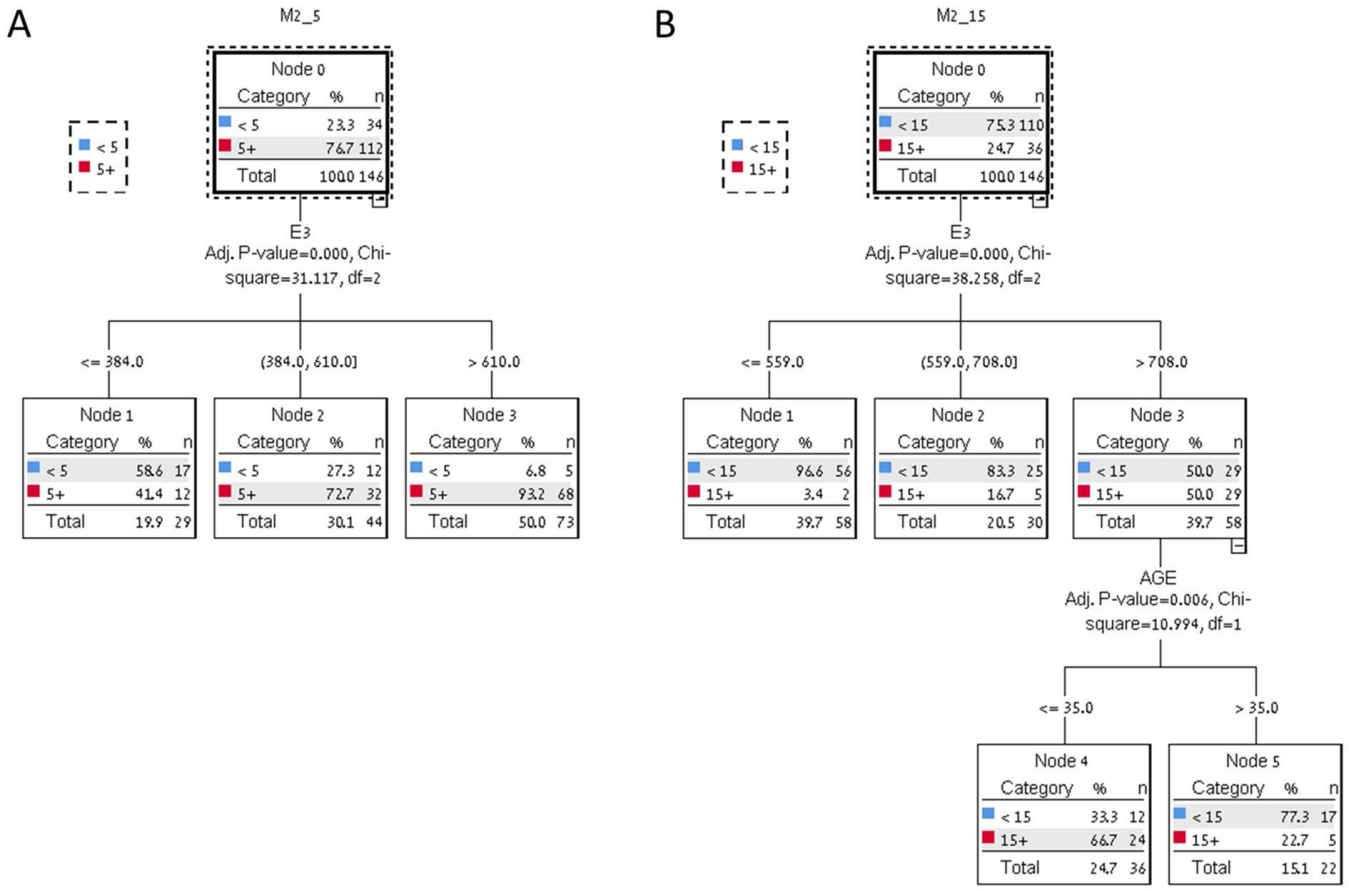
CHAID analysis Tree diagram to predict the retrieval ≤ 5 M2 oocytes (**A**) based on GAST E3 values (pmol/l) and > 15 M2 oocytes (**B**) based on GAST E3 values (pmol/l) and age (years).

Decision tree CHAID (Chi-square automatic interaction detector) analysis was another technique that can be used to determine suitable cutoff value for GAST E3 (Fig. 2). We show that patients with GAST E3 ≤ 385 pmol/l are at 58.6% risk to obtain less than 5 oocytes suitable for freezing and GAST E3 > 610 pmol/l are 93.2% likely to obtain more than 5 oocytes regardless of age.

Patients with GAST E3 ≤ 559 have only 3.4% chance to freeze over 15 oocytes, whereas patients younger than 35 with GAST E3 > 708 pmol/l have 66% chance for freezing over 15 oocytes. Patients over 35 years old in this group only 22.7% chance to freeze over 15 oocytes.

## Discussion

In this prospective study, we demonstrated that the response to GnRHa administration in early follicular phase, prior to stimulation, could predict ovarian response and fertility preservation outcome in antagonist cycles. It can be appreciated that maximizing oocyte yield for cryopreservation is important, given that it is also a predictor of live birth rate^3^. It was shown by Cubo et al. that in patients undergoing fertility preservation the cumulative live birth rate (CLBR) is age dependent. Patients younger than 35 require approximately 15 oocyte to achieve 69.9% CLBR whereas older patients can achieve 49.6% CLBR from 20 cryopreserved oocytes^3^. Birth rate in patient undergoing fertility preservation over 40 years old was very low^3^. The average age in our study population was 35 years old but 43% of the patients were older. Nonetheless, all the patients were under 40 years old.

The population of patients seeking social fertility preservation is unique due to the fact they apparently have no infertility issues and usually were not treated for infertility. Thus, the assessment of their fertility potential is usually based on endocrine tests such as AMH, FSH levels and ultrasound evaluation of antral follicle count. Based on these parameters the patients are consulted and given stimulation protocols. Dynamic tests for ovarian reserve such as EFFORT (Exogenous follicle stimulating hormone ovarian reserve test), GAST and CCCT (Clomiphene Challenge Test) were used mainly during ART treatments for achieving pregnancy (reviewed in^8^). In most settings the dynamic tests were conducted separately from the treatment cycle and are less and less used in clinical practice. Some researchers used GAST as part of a short GnRH agonist protocol^13^.

In this study we utilize for the first time GAST, a dynamic test for ovarian reserve, as part of short antagonist treatment protocol in the context of fertility preservation. We found that the estradiol response ratio to single dose of 0.2 mg GnRH agonist is strongly correlated with treatment outcome as measured by the number of mature M2 oocyte good for cryopreservation. This observation is probably related to the number of small antral follicles that express enough FSHR and be recruited. Since it can be assumed that small antral follicles secrete similar amount of estradiol, a stronger response can represent more follicles^15^.

Several studies in the past evaluated the ovarian response to GnRH agonist (GAST assay). The studies used different formulations of GnRH agonist, different doses and protocols. Earlier studies measured the response pattern to GnRHa over several days of stimulation^9,10,16^. Some studies measured the levels of different hormones such as Estradiol, FSH and inhibin B^13,16–18^. We observed that a single measurement using the absolute value of estradiol as well as ratio of estradiol rise are a good marker for ovarian response.

We observed that GAST and AFC are more predictive tests than basal day 3 FSH levels. This can be explained by the fact that these tests represent the current cycle, ovarian reserve potential compared to day 3 FSH which is not a direct measurement of the follicular activity or number. GAST has the advantage that it does not require ultrasound visualization, is not operator dependent and has proven efficacy in earlier studies^5^. We found some benefit in combining the values of AFC and E3 GAST in predicting poor ovarian response (ROC) however the increase was not significant. Perhaps combining GAST with more ovarian reserve tests such as AMH would provide even better power of prediction.

A limitation of this study was the fact that we omitted AMH results due limited available for some of the patients. However, it was shown in the past that AFC is a comparable marker to predict ovarian response^19^. Thus, we used AFC in conjunction with day 3 FSH levels and compared them to GAST.

Lashen et al., utilized GAST to predict over-response in a short agonist IUI treatment. They calculated the estradiol ratio after one day of GnRH agonist treatment and determined an estradiol ratio cutoff of 3.7 (sensitivity 0.52, PPV 0.55 NPV 0.55) to predict more than 3 mature follicles. However, that study was not carried out in IVF patients without known infertility diagnosis and the average age and amount of gonadotropins used were lower than in our study^17^.

In our group of patients there was no correlation between the patient's age and the number of M2 oocytes that were obtained. This can be explained by the fact that our study population consisted mostly patient under 37 years old in which the effect of the age is less significant. We did observe using CHAID analysis that among the sub group of high responders a higher proportion of the young patients under 35 years old was able to freeze over 15 oocytes. This difference not demonstrated in the analysis of the entire population of the study probably due to the size of the group.

GAST can be another important tool for the clinician when consulting the patient regarding the ovarian reserve. Using automatic classification CHAID analysis we found that a test values of GAST E3 of over 610 pmol/l can be reassuring since over 93% of the patients had more than 5 oocytes. A value of over 708 pmol/l was observed in 50% of those freezing over 15 oocytes—nearly identical to the cutoff deducted from AUC analysis. Low GAST might suggest postponement of cryopreservation to a cycle with a higher response to follicular GnRHa, or increase dosage, but this was not tested in this study.

Hendriks et al. interestingly, assayed GAST response a patient in two different cycles but not as part of a treatment protocol. They concluded that the assay is good in predicting poor responders with lower pregnancy potential. However, all their patients were treated in the long agonist downregulation protocol using a fixed starting dose of 150U of gonadotropins. The current definition of poor responders usually refers to treatment dose of at least 300U of gonadotropins per day^18^. The authors observed certain GAST response cycle variability in some of the patients and argued that the fluctuation can only be attributed to chance variation. However, in contrast to their conclusion, it was shown that there is some cycle to cycle variation of hormonal variables and oocyte yield^20^ not explained by baseline AFC and AMH^21^ which may attribute to GAST individual cycle results.

The addition of an agonist at the beginning of the stimulation adds a flare-up effect that might be beneficial to the follicular recruitment; also, we used the flareup to predict suboptimal response to GnRH agonist trigger, which is especially relevant in fertility preservation cycles, since the use of hCG triggering is usually avoided to prevent OHSS^14^.

In conclusion, our results suggest that GAST test can be used to predict the current cycle potential yield. since several patients with apparently normal ovarian reserve as determined be Day 3 FSH levels had abnormal GAST test and eventually low number of mature oocytes retrieved. In those patients, one might consider to delay treatment to the next month to repeat GAST due to cycle variability.

Future studies should test whether adjustment of gonadotropins dosage according to GAST follicular challenge test could improve oocyte yield or avoid hyperstimulation in non- cryopreservation cycles.

## Methods

### Study design

We recruited all women that underwent fertility preservation in our tertiary university affiliated medical center.

Informed consent was obtained from all subjects prior to the start of the study protocol.

The experiment protocol was approved by Shamir Medical center IRB committee (0304-20-ASF, 13/06/2021) and was registered in ClinicalTrials.gov (NCT04973969, 22/07/2021).

All women received antagonist protocol. The dosage and Gonadotropins (GT) used were chosen by the treating physician based on age, hormonal profile and previous history if existed.

On day 2 to menstrual cycle, blood tests were drawn (basal Estradiol/E2, basal FSH/FSH2, basal LH/LH2, Progesterone/P2) and ultrasound (US) was performed to confirm no ovarian cysts existed and AFC was documented. On that evening, the women were instructed to inject 0.2 mg GnRH agonist (Decapeptyl, FERRING) and arrive for repeated blood workup 10–12 h later at the next morning (GAST E3, FSH3, LH3, P3), followed by a flexible antagonist protocol, with administration of antagonist when follicles’ diameter was above 12 mm or estradiol levels reach 1000 pmol/l or more^22^. The gonadotropin dose and type (recombinant FSH/LH or highly purified human menopausal gonadotropin) used in the study was decided by the treating physician according to clinical parameters such as age and predicted ovarian reserve. The dose range and average daily dose is described in Table 1. Oocyte maturation was achieved once the mean diameter of three or more follicles was 18 mm with 0.2 mg GnRH agonist (Decapeptyl, FERRING) and LH was checked 24 h later. Oocyte retrieval was scheduled 36 h after final oocyte maturation trigger.

We documented demographic characteristics (age, BMI), stimulation characteristics (GT used, total dosage of GT, stimulation days, hormonal profile on each visit. The main outcome measure was the number of Mature oocytes (M2) suitable for cryopreservation. We also documented the total oocytes retrieved and immature oocytes (GV, M1). The sample size was calculated to identify a weak correlation (r = 0.3) using significance level of 5% with power level of 80%. The sample size needed was 85 cases.

All methods were performed in compliance with the relevant guidelines and regulations.

### Statistics

SPSS software was used for statistical analysis (IBM Corp. Released 2021. IBM SPSS Statistics for Windows, Version 28.0. Armonk, NY: IBM Corp). All statistical tests were two-sided and p < 0.05 was considered a statistical significance.

Categorial variables were described as frequencies and percentages. Distribution of continuous variables was evaluated and reported as median and interquartile range (IQR). Spearman correlation coefficient was used to asses association between continuous variables and Mann—Whitney test was used to compare continuous variables between categories and paired T test for paired samples.

Patients were divided according to the number of M2 oocytes that were obtained. Under 5 oocytes and over 15 oocytes.

The area under the ROC curve (AUC) was used to evaluate the discrimination ability between groups (less than 5 or more than 15 M2 oocytes).

CHAID (Chi-square automatic interaction detector) method was used to create a decision tree to determine optimal cutoff values and parameters (GAST E3, age) to discriminate between the groups^23^. The procedure automatically excludes any variable whose contribution to the final model is not significant, and a decision tree is obtained. A step-by-step methodology was followed to select the independent variables. The level of significance for the splitting nodes was 0.05.

## Data Availability

The data underlying this article will be shared upon reasonable request to the corresponding author. The subjects in this trial have not concomitantly been involved in other randomized trials. Data regarding any of the subjects in the study has not been previously published. Data will be made available to the editors of the journal for review or query upon request.
